# Resting-state functional connectivity does not predict individual differences in the effects of emotion on memory

**DOI:** 10.1038/s41598-022-18543-8

**Published:** 2022-08-25

**Authors:** Dona Kandaleft, Kou Murayama, Etienne Roesch, Michiko Sakaki

**Affiliations:** 1 School of Psychology and Clinical Language Sciences, University of Reading, Reading, UK; 2 Centre for Integrative Neuroscience and Neurodynamics, University of Reading, Reading, UK; 3 Hector Institute for Education Sciences and Psychology, University of Tübingen, Tübingen, Germany; 4 Research Institute, Kochi University of Technology, Kochi, Japan

**Keywords:** Cognitive neuroscience, Emotion

## Abstract

Emotion-laden events and objects are typically better remembered than neutral ones. This is usually explained by stronger functional coupling in the brain evoked by emotional content. However, most research on this issue has focused on functional connectivity evoked during or after learning. The effect of an individual’s functional connectivity at rest is unknown. Our pre-registered study addresses this issue by analysing a large database, the Cambridge Centre for Ageing and Neuroscience, which includes resting-state data and emotional memory scores from 303 participants aged 18–87 years. We applied regularised regression to select the relevant connections and replicated previous findings that whole-brain resting-state functional connectivity can predict age and intelligence in younger adults. However, whole-brain functional connectivity predicted neither an emotional enhancement effect (i.e., the degree to which emotionally positive or negative events are remembered better than neutral events) nor a positivity bias effect (i.e., the degree to which emotionally positive events are remembered better than negative events), failing to support our pre-registered hypotheses. These results imply a small or no association between individual differences in functional connectivity at rest and emotional memory, and support recent notions that resting-state functional connectivity is not always useful in predicting individual differences in behavioural measures.

## Introduction

Emotional events are typically remembered better and more vividly relative to neutral ones^1–3^. This emotional enhancement effect has been found in laboratory studies^4–8^ as well as autobiographical memory^9^. Previous research has suggested that individual differences in this emotional memory enhancement effect may have important consequences on wellbeing and psychopathology. For example, the enhancement effects of emotion on memory are considered to result in spontaneous and intrusive recollection of traumatic memories^10^. Likewise, one’s tendency to preferentially remember negative information is frequently present in psychopathological conditions, including depression and anxiety, and is associated with symptom severity^11^. This negative memory bias has also been associated with smaller hippocampal grey and white matter volume^12^, which is in turn associated with major depression^13^. In contrast, one’s tendency to preferentially remember positive over negative information is referred to as “positivity bias”, and is often associated with better emotional wellbeing in old age^14,15^. In the current study, we tested whether such individual differences in the emotional enhancement effects of memory can be predicted by resting-state functional connectivity in the brain. Functional connectivity (FC) refers to the strength of connections between brain areas that share functional properties. We distinguish task-induced FC in response to a stimulus, from resting-state FC, which reflects the connectivity of an individual at rest.

The brain mechanisms behind the emotion-induced enhancement effects in memory have been intensively studied in task-fMRI studies, where researchers examined blood oxygen level dependent (BOLD) signals obtained while participants encoded emotional vs. non-emotional information. Meta-analyses based on these studies reported that the enhancement of emotional memory is associated with increased activation in the amygdala, hippocampus, and regions in the ventral visual stream during the encoding of emotional items^16,17^. In addition to the activation level, increased task-induced FC across the amygdala, hippocampus and the prefrontal cortex (PFC) during encoding of emotional items is associated with enhanced memory for emotional compared with neutral items^18–22^. Previous studies also extended their focus to FC after learning (i.e., during consolidation)^23,24^. Stronger FC between the amygdala and visuosensory areas after learning was associated with the negative memory bias in memory, whereas stronger FC between the amygdala and anterior cingulate after learning was associated with the positivity bias in memory^24^.

In contrast, it has been less clear whether resting-state FC before learning predicts individual differences in emotional memory. Resting-state FC refers to the temporal correlation in activity between regions that are not actively engaged in any task, and is considered to reflect the brain’s functional and structural connectivity^25^. Individual differences in resting-state FC have been used to predict individual differences in brain activation during various tasks, including working memory, language tasks, emotion recognition, and interpreting social interactions^26^. Research on memory has further demonstrated an association between memory performance for neutral items and resting-state FC of the MTL^27,28^ and the default mode network (DMN) which has been implicated in age-related cognitive decline^29,30^.

In addition, recent advances in machine learning have allowed researchers to identify and study complex data models, that can be used to predict individual differences from a wide range of behavioural and cognitive measures^31^. Studies implementing such analyses found that resting-state FC predicts behavioural measures including attention span^32,33^, decision-making strategies^34^, intelligence^35,36^, motor skills learning^37^ and personality^36^, acting as a behavioural “fingerprint”^35^.

In contrast, few studies have investigated whether resting-state FC predicts individual differences in emotional memory. On the one hand, FC during rest resembles the FC observed during a task^38,39^ and previous findings support an association between emotional memory enhancement effects and FC during rest before^22,40^ or after encoding^23,24^. Therefore, it is reasonable to hypothesise that whole-brain resting-state FC is predictive of individual differences in emotional memory. On the other hand, recent evidence emphasised that robust cognitive tasks may not always yield reliable inter-individual measures^41^. Similar low reliability was also reported for the emotional enhancement effect in memory, despite robust and strong group-wise effects for better memory for emotional rather than neutral items^42^. Therefore, even though resting-state FC has a relatively high temporal reliability^35,43^, resting-state FC may not be able to reliably predict emotional memory enhancement effects.

We investigated whether resting-state FC predicts emotional memory using a large database—the Cambridge Centre for Ageing and Neuroscience (Cam-CAN)—that includes emotional memory scores, structural and functional MRI (fMRI) scans of 303 individuals of ages 18–87 years^44,45^. In the Cam-CAN project, participants completed an emotional memory task (in a different session from the MRI session), where they learned neutral objects superimposed onto emotionally positive, neutral, and negative backgrounds^46^. Consistent with the emotion induced enhancement effect observed in the literature, participants had a better memory for objects learned with positive or negative backgrounds than objects learned with neutral backgrounds (Table 1). Based on this task, we created two continuous measures of emotional memory: (a) better memory for positive and negative information than neutral information (the emotional enhancement effects) and (b) preferential memory for positive rather than negative information (the positivity bias). Our study also attempts to predict age and intelligence from resting-state FC; these latter analyses served as control checks to ensure that our method and data can replicate previous findings^35,36,47^.

**Table 1:** The mean and standard deviation of memory scores for participants across all ages (18–87 years old).

Memory type	Negative		Positive		Neutral		*F*(2, 604)	Partial η^2^	*p*
	M	SD	M	SD	M	SD			
Object memory	2.64^a^	0.79	2.70^b^	0.74	2.58^c^	0.76	18.6	0.058	< 0.001
Associative valence memory	1.59^a^	0.75	1.19^b^	0.63	0.90^c^	0.62	424.1	0.584	< 0.001
Background memory	0.16^a^	0.09	0.14^b^	0.08	0.08^c^	0.06	114.2	0.274	< 0.001

The object memory refers to participants’ memory performance for neutral objects learned with positive, negative or neutral backgrounds. The associative valence refers to memory for whether each neutral object was associated with a positive, negative or neutral background. The background memory concerns memory performance for the details of the background image associated with each neutral object^46^. The d′ scores are used for the object and associative valence memory. The proportion of correct gist memories is used for the background memory measure. Means with different subscript letters were statistically different (*p* < 0.05) according to pairwise comparisons with Bonferroni correction.

We preregistered the above hypotheses and analysis pipelines, which are accessible at https://osf.io/untzm. Following an analysis pipeline previously used to predict individual differences in personality and intelligence from resting-state FC^36^, we used regularised linear regression to predict the emotional enhancement effect and the positivity bias in memory from whole-brain resting-state FC. The brain was parcellated into 268 nodes obtained from Shen et al.^48^. Seven nodes were excluded from the analysis due to missing data, therefore comprising a total number of predictors of 33,930 connections. Due to the expected collinearity and large number of predictors, we used common parameter regularization techniques to avoid over-fitting of the data models. Specifically, we used Elastic Net penalization, which combines ridge (L1) and lasso (L2) penalization schemes. Ridge regularization adds a Gaussian prior to the parameters of the model. Lasso penalization provides an upper bound to the parameter, while creating opportunities to reduce the number of predictors altogether. Additionally, we used leave-one-out cross-validation to train and test the models, and permutation testing to compute a *p*-value when *R*^2^ showed a positive relationship (permutation analyses were not run when *R*^2^ was negative because negative *R*^2^ means that the models performed poorly).

## Results

The analysis procedure for the main analyses (where we predicted the emotional enhancement effects, the positivity bias and intelligence from resting-state FC across all participants) was preregistered, and the scripts used are publicly available (https://osf.io/bm98y).

### Behavioural results

A composite score for intelligence was computed from the four subsets of Cattell through principal component analysis. The derived factor explained 67.8% of the total variance, and had loadings ranging from 0.81 to 0.84 with the four Cattell subsets. As reported in the original paper about the dataset^46^, participants showed better object memory for positive and negative backgrounds than neutral backgrounds (Table 1). We further computed a measure of the emotional enhancement effect variable by subtracting object memory performance in the neutral condition from the average memory performance in the positive and negative conditions. We also created another measure of positivity bias by subtracting object memory performance in the negative condition from memory performance in the positive condition.

From the original dataset, we used 303 participants (Table 2)—all the participants in the database who completed the resting-state fMRI, emotional memory task, and the intelligence test. The data included 261 nodes, as one or more of those seven nodes—located in the left and right temporal lobes—were missing for 45 participants. We computed the following exploratory correlations analyses as quality checks. Older individuals performed more poorly on the intelligence score than younger individuals, *r*(301) = − 0.63, *p* < 0.001. In contrast, age was not significantly correlated with the positivity bias, *r*(301) = 0.11, *p* = 0.06, nor with the emotional enhancement effect of memory, *r*(301) = 0.01, *p* = 0.89. There were no significant gender differences in intelligence, *t*(301) = 1.77, *p* = 0.08, the positivity bias, *t*(301) = − 1.40, *p* = 0.17, or in the emotional enhancement effect, *t*(301) = 0.27, *p* = 0.79.

**Table 2:** Characteristics of participants across ages (18–87 years), younger adults (18–40 years), middle-aged (41–60 years), and older adults (61–87 years).

	All	Younger adults	Middle-aged	Older adults
N	303	85	98	120
Age	54.3 (18.1)	31.8 (5.8)	50.7 (5.8)	73.3 (7.0)
Gender (males:females)	155:148	44:41	48:50	63:57
Intelligence	0.00 (1.00)	0.73 (0.66)	0.23 (0.73)	− 0.71 (0.93)
**Education level (N)**				
Degree	191	68	65	58
A-Levels	55	9	19	27
GCSE/O-Level	36	8	12	16
None	20	0	2	18

Intelligence refers to the composite score of intelligence on the fluid intelligence test. Information about education level was missing for one participant in the older adults age group. All data are specified as mean (sd) unless otherwise specified.

### Preregistered predictive modelling

We followed a strategy first described by Dubois et al.^36^. Before running prediction analyses, preprocessing and denoising pipelines were run on the resting-state images. The pipelines included (1) applying motion correction, (2) registration to the standard Montreal Neurological Institute (MNI) brain template, (3) detrending the white matter and cerebrospinal fluid through removing temporal drifts with third-degree Legendre polynomial regressors, (4) regressing out mean signals of the white matter and cerebrospinal fluid from the grey matter signal, (5) regressing out motion parameters from the whole brain, (6) removing high-frequency noise by applying a low-pass filter (1 TR which is 1970 ms in this study), (7) detrending the grey matter signal though removing temporal drifts with third-degree polynomial Legendre regressors, and (8) regressing out global signals from the whole brain signal.

**Figure 1:**
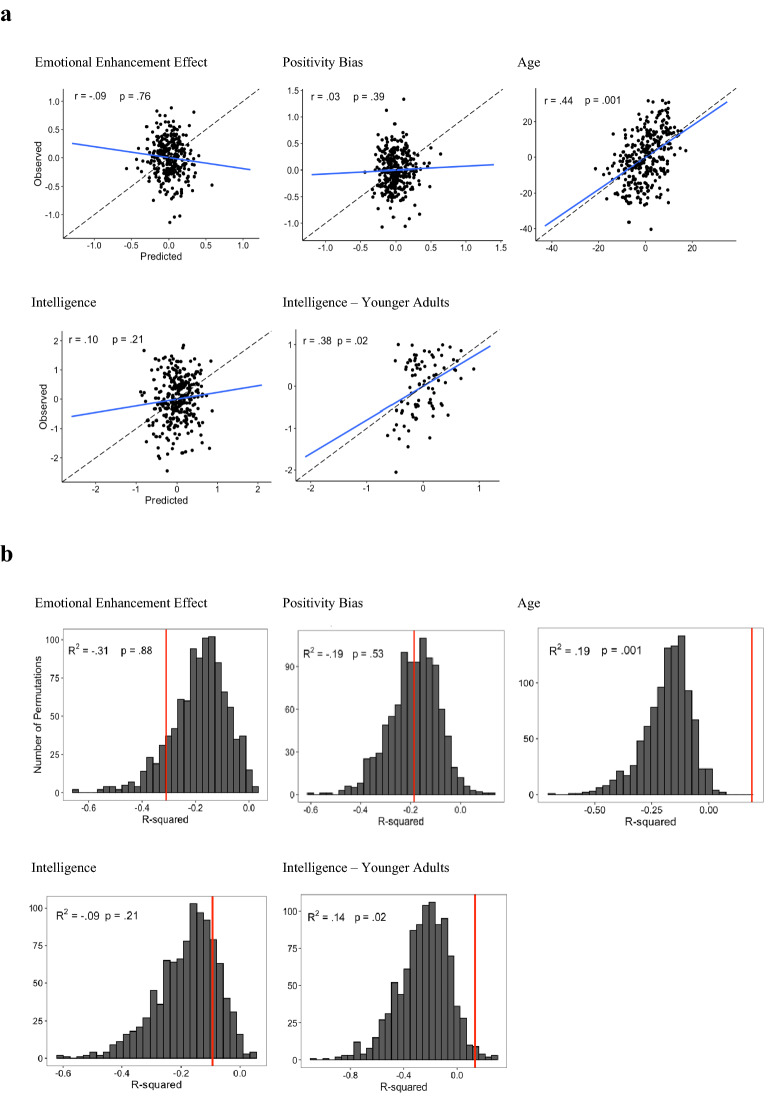
The prediction performance of the models for emotional enhancement effect, positivity bias, age, intelligence, and intelligence for younger adults only. (**a**) Scatter plots showing demeaned and deconfounded observed values versus those predicted by the models. Pearson’s correlation and the one tailed *p* value of the correlation obtained from permutation are shown on the graph. The best fitting line is displayed in blue. Slopes closer to 1 (dotted line) show good prediction^36^. (**b**) The distribution of the permutation models’ *R*^2^ (in grey), which is the null distribution. The model’s *R*^2^ are shown in red. The models’ *R*^2^ and one-tailed *p* value obtained from permutation are displayed on the figures.

Prediction analyses began with filtering, whereby only edges with correlations of *p*-value < 0.01 with the predicted variable were included in the subsequent analyses. We used Elastic Net models with a high ratio of ridge (0.9) and tuned the models’ alpha parameter through a grid search. Analyses were run to predict the emotional enhancement effect in memory, the positivity bias in memory, age, and intelligence from the connectivity matrix. The control variables were age, gender, handedness and intelligence (unless they are the predicted variable) which were regressed out from the predicted variables (see Supplementary Table 1 for analyses including motion as a control variable). The models were trained in leave-one-out cross validation. We ran one thousand permutations of the data, which allowed us to calculate one-tailed *p*-values for each model that returned positive *R*^2^. Results are shown in Fig. 1. The models predicting the emotional enhancement effect and the positivity bias performed poorly, demonstrating negative correlations between the predicted and observed values (Table 3; Fig. 1). The model predicting intelligence also performed poorly and did not achieve a significant correlation between predicted and observed values (Table 3; Fig. 1).

**Table 3:** Model prediction results when including participants across all age groups (18–87 years old).

Dependent variable	*r*	*R*^2^	nRMSD	*p*	Predictive edges (N)
**Main analyse**					
Emotion enhancement effect	− 0.09	− 0.31	1.14	0.88	161
Positivity bias	0.03	− 0.19	1.09	0.53	212
Intelligence	0.09	− 0.09	1.05	0.21	522
**Exploratory analysis**					
Age	0.44	0.19	0.90	0.001	5555

For all variables, we used Elastic Net, with ridge-lasso ratio = 0.01. The models were trained using leave-one-out cross-validation. *p*-values were calculated as the number of permutations with lower *R*^2^ divided by 1000. The emotion enhancement effect refers to the degree to which neutral objects were learned better when they were paired with emotional rather than neutral background images. The positivity bias represents the degree to which objects paired with positive backgrounds were remembered better than those paired with negative backgrounds. The number of predictive edges represents the average number of edges that were included after filtering and regularisation across all folds.

### Exploratory analyses

As described in the previous section, our pre-registered analyses failed to predict our two emotional memory measures from resting-state FC. We also failed to replicate previous findings showing that resting-state FC can predict intelligence. We therefore ran a series of unplanned exploratory analyses to identify when resting-state FC predicts behavioural measures. First, we ran an exploratory analysis to test if we can replicate previous findings that one’s chronological age is predicted by resting-state FC^47,49^. The model obtained good prediction, achieving strong correlation between predicted and observed values, *r*(301) = 0.44 (Table 3; Fig. 1), suggesting that resting-state FC is predictive of an individual’s age.

#### Analysis for each age group

Next, we performed an exploratory analysis after splitting the sample into three age groups: younger (aged 18–40 years; N = 85), middle-aged (aged 41–60 years; N = 98) and older adults (aged 61 years and over; N = 120) given that previous studies on intelligence and resting-state FC primarily focused on younger adults^35,36^, whereas our participants included those aged between 18 and 87 years. Note that past studies also showed the non-linear effects of age; suggesting that older adults may rely on a different set of regions (relative to younger adults) to perform the same task^50,51^. To test the possibility that the emotional enhancement effect, the positivity bias and intelligence are successfully predicted after splitting participants into separate age groups, the analyses were repeated separately for each age group, with the same methodology as the whole-sample analyses described above. The model successfully predicted intelligence in younger adults, but not for middle-aged or older adults (Table 4). However, the model still failed to predict the emotional memory enhancement effect and the positivity bias across all groups (Table 4).

**Table 4:** Model prediction results of participants for each age group.

Dependent variable	Group	*r*	*R*^2^	nRMSD	*p*
Emotion enhancement effect	Younger adults	− 0.13	− 0.33	1.15	0.92
	Middle-aged	− 0.08	− 0.24	1.11	0.77
	Older adults	− 0.17	− 0.31	1.15	0.87
Positivity Bias	Younger adults	− 0.23	− 0.49	1.22	0.94
	Middle-aged	− 0.15	− 0.41	1.19	0.75
	Older adults	0.16	− 0.06	1.03	0.14
Intelligence	Younger adults	0.38	0.14	0.93	0.02
	Middle-aged	0.17	− 0.05	1.03	0.14
	Older adults	0.00	− 0.17	1.08	0.38

For all analyses, we used Elastic Net, with ridge-lasso ratio = 0.01. The models were trained using leave-one-out cross-validation. *p* values were calculated as the number of permutations with lower *R*^2^ divided by 1000.

#### Other emotional memory measures

Results presented so far concerned memory accuracy for neutral objects that were superimposed on negative, neutral or positive images (so called ‘object memory’). Yet the CamCAN study tested three types of memory: object, associative valence and background memory^45^. While the effects of valence on this object memory measure were significant, they were relatively small^46^; which may have resulted in our failure to predict the emotional memory enhancement effects using resting-state FC. To address this issue, we applied the same analysis method again to the two other types of memory in the Cam-CAN dataset: associative valence memory and background memory. The associative valence memory measure concerns whether each correctly-recalled neutral object was associated with a positive, negative or neutral background and showed stronger effects of valence compared with object memory^46^ (see Table 1). In contrast, the background memory concerns participants’ gist memory for contents of the positive, negative and neutral background images. This gist background memory also showed significant effects of valence, such that participants had a better background memory for the negative than the positive condition, which was better than the neutral condition (see Table 1).

As done in the object memory, we obtained the emotional enhancement effect and the positivity bias for both the associative and background memory measures and ran the same set of analyses. But the models derived from resting-state FC could not significantly predict either the emotional enhancement effect or the positivity bias even in these measures (Table 5). We also ran the same analysis after splitting participants into three age groups, but the models could not predict the emotional enhancement effect or positivity bias in any group.

**Table 5:** Model prediction results for other memory measures.

Dependent variable	Ages	*r*	*R*^2^	nRMSD
Emotional enhancement effect—associative valence memory	All ages	0.00	− 0.14	1.07
	Younger adults	0.10	− 0.14	1.07
	Middle-aged	− 0.27	− 0.47	1.21
	Older adults	0.05	− 0.17	1.08
Positivity bias—associative valence memory	All ages	0.20	0.00	1.00
	Younger adults	0.20	− 0.03	1.02
	Middle-aged	− 0.12	− 0.22	1.10
	Older adults	0.05	− 0.17	1.08
Emotional enhancement effect—background memory	All ages	− 0.15	− 0.33	1.15
	Younger adults	− 0.15	− 0.43	1.20
	Middle-aged	− 0.05	− 0.35	1.16
	Older adults	− 0.06	− 0.27	1.13
Positivity bias—background memory	All ages	− 0.19	− 0.39	1.18
	Younger adults	− 0.11	− 0.27	1.13
	Middle-aged	− 0.18	− 0.41	1.19
	Older adults	0.15	− 0.11	1.05

### Robustness check

To check that the results were not specific to the analysis method we used, we ran a series of analyses with other methods and parameters. First, we ran the same set of analyses while changing the lasso-to-ridge ratio from 0.01 to an automatic selection in threefold nested cross-validation among 6 ratios (0.1, 0.5, 0.7, 0.9, 0.99, 1), to check whether the quality of parameter regularization would impact the results^52–56^. The results showed similar patterns; resting-state FC successfully predicted age and intelligence in younger adults but none of the other variables (Table 6).

**Table 6:** Prediction results of alternative models.

Dependent variable	Ages	Model	*r*	*R*^2^	nRMSD
Object emotion enhancement effect	All ages	Filtering threshold = 0.01, Elastic Net, fixed L1, LOOCV	− 0.09	− 0.31	1.14
	All ages	Filtering threshold = 0.01, Elastic Net, fixed L1, 10− Fold CV	0.03	− 0.15	1.07
	All ages	Filtering threshold = 0.01, Elastic Net, tuned L1, LOOCV	− 0.09	− 0.33	1.15
	All ages	Filtering threshold = 0.01, Random Forest, LOOCV	0.04	− 0.10	1.05
	All ages	Filtering threshold = 0.05, Elastic Net, fixed L1, LOOCV	0.20	− 0.05	1.02
Object positivity bias	All ages	Filtering threshold = 0.01, Elastic Net, fixed L1, LOOCV	0.03	− 0.19	1.09
	All ages	Filtering threshold = 0.01, Elastic Net, fixed L1, 10-Fold CV	0.02	− 0.25	1.12
	All ages	Filtering threshold = 0.01, Elastic Net, tuned L1, LOOCV	0.03	− 0.22	1.10
	All ages	Filtering threshold = 0.01, Random Forest, LOOCV	0.01	− 0.13	1.07
	All ages	Filtering threshold = 0.05, Elastic Net, fixed L1, LOOCV	0.02	− 0.23	1.11
Intelligence	All ages	Filtering threshold = 0.01, Elastic Net, fixed L1, LOOCV	0.09	− 0.09	1.05
	All ages	Filtering threshold = 0.01, Elastic Net, fixed L1, 10− Fold CV	0.06	− 0.14	1.07
	All ages	Filtering threshold = 0.01, Elastic Net, tuned L1, LOOCV	0.14	− 0.07	1.04
	All ages	Filtering threshold = 0.01, Random Forest, LOOCV	0.14	− 0.04	1.02
	All ages	Filtering threshold = 0.05, Elastic Net, fixed L1, LOOCV	− 0.16	− 0.15	1.07
Age	All ages	Filtering threshold = 0.01, Elastic Net, fixed L1, LOOCV	0.44	0.19	0.90
	All ages	Filtering threshold = 0.01, Elastic Net, fixed L1, 10− Fold CV	0.43	0.18	0.91
	All ages	Filtering threshold = 0.01, Elastic Net, tuned L1, LOOCV	0.41	0.15	0.92
	All ages	Filtering threshold = 0.01, Random Forest, LOOCV	0.32	0.10	0.95
	All ages	Filtering threshold = 0.05, Elastic Net, fixed L1, LOOCV	0.46	0.21	0.89

Fixed L1 is ridge-lasso ratio = 0.01. Tuned L1 refers to the procedures where L1 was chosen using a threefold nested cross-validation from the values: 0.1, 0.5, 0.7, 0.9, 0.99, 1. LOOCV refers to leave one-out cross validation. Random forest models tune the maximum depth parameter from the 5 values: 5, 10, 20, 40, 50, using a nested threefold nested cross-validation. Filtering threshold refers to the maximum *p* value of the correlation between individual edges and the predicted variable that was required for edges to be included in the prediction analysis.

The same set of analyses were also run again but using tenfold cross-validation instead of leave-one-out cross-validation, as k-fold cross-validation may show higher robustness than leave-one-out cross-validation^57^. Once again, age was successfully predicted. Likewise, intelligence in younger adults was predicted by resting-state FC. Nevertheless, none of the rest of variables were predicted by resting-state FC (Table 6).

Thirdly, the analyses were run using a Random Forest Regressor instead of a linear regression. Leave-one-out cross-validation was used, allowing for a maximum depth among 5 values (5, 10, 20, 40, 50), similar to parameters used in previous studies^58–61^. The results were again similar, showing good prediction for age and intelligence in younger adults, but not the other variables (Table 6).

Fourthly, we also changed the edge filtering threshold. Across all the analyses described so far, we applied the edge filtering threshold of 0.01 to include only edges that correlated with the predicted behavioural measure with *p* value < 0.01. To test the effects of this filtering threshold, we ran exploratory analyses using filtering thresholds of 0.02, 0.03, 0.04 and 0.05 for intelligence in younger adults, a behavioural domain where we saw one of the strongest prediction results (Supplementary Table 2). This exploratory analysis showed that intelligence in younger adults was best predicted by a filtering threshold of *p* = 0.05, resulting in r = 0.37, R-square = 0.12, and nRMSD = 0.94. Based on this analysis, the main analyses (collapsed across age groups) were performed again for all prediction models using this new filtering threshold (p = 0.05). However, the results were also similar; the resting-state FC predicted age but not emotional memory measures nor intelligence (Table 6).

#### Including all edges

As described earlier, we excluded data from seven nodes given that 45 participants did not have data from one of these nodes. The excluded nodes are located in the left and right temporal lobes. To ensure that the results are not affected by our exclusion of these nodes, the main analyses were conducted again using the preregistered methods (edges filtering at p = 0.01, Elastic Net, L1 = 0.01, leave one-out cross validation) while including all the edges; this resulted in 258 participants without the 45 participants who had missing data in these edges (N = 13 younger adults; N = 15 middle-aged adults; N = 17 older adults). Once again, the analyses showed that only age was significantly predicted by resting-state FC (Table 7).

**Table 7:** Prediction results when including all edges (N = 258 participants).

Dependent variable	Ages	*r*	*R*^2^	nRMSD
Emotion enhancement effect	All ages	0.01	− 0.20	1.09
Positivity bias	All ages	− 0.28	− 0.50	1.23
Intelligence	All ages	0.07	− 0.13	1.06
Age	All ages	0.40	0.16	0.92

For all analyses, all nodes were included. Forty-five participants were excluded due to missing data in one or more nodes. For all analyses, we used Elastic Net, with ridge-lasso ratio = 0.01. The models were trained using leave-one-out cross-validation.

## Discussion

In this study, we examined whether resting-state FC predicts individual differences in the emotional enhancement effect in memory, the positivity bias in memory, intelligence and age. Neither the emotional memory enhancement effect, nor the positivity bias was significantly predicted by resting-state FC. In contrast to these measures of emotional memory, models derived from resting-state FC successfully predicted chronologic age, replicating previous findings^47,49^. These results suggest that the methods used in this study were able to predict behavioural phenotypes based on resting-state FC. Yet, contrary to our prediction (preregistered), intelligence was not predicted from resting-state FC when participants of all ages were included.

To check whether the failure to predict intelligence or emotional memory measures using resting-state FC was due to the wide age range of participants, we split participants into three groups: younger (18–40 years), middle-aged (41–60 years), and older adults (61–87 years). Previous studies suggest that resting-state FC patterns undergo a nonlinear trajectory with age, such as increasing FC within DMN during late adulthood before its rapid decline after age 74^29^. In addition, age-related compensatory recruitment of the prefrontal cortex can result in age-related shifts in brain regions responsible for tasks relevant to intelligence^50^. However, neither the emotional memory enhancement effect nor the positivity bias was predicted by the models in any age groups. The only exception was intelligence in younger adults; when including only younger adults as done in past studies^35,36^, resting-state FC successfully predicted individual differences in intelligence.

Importantly, even after splitting participants into three age groups, resting-state FC did not predict the two emotional memory measures in any age groups. These results suggest that the predictive power of resting-state FC is lower for emotional memory measures than for intelligence. The results could also suggest that the utilized emotional memory measures are not appropriate or reflective of a reliable effect. These results are in line with those from past studies on resting-state FC. For example, a recent study failed to replicate past findings in predicting habitual use of emotion regulation strategies from resting-state FC^62^. Another study showed that resting-state FC predicts working memory, but not executive control, language, or verbal memory performance in older adults^63^. Similarly, in Dubois et al.^36^, resting-state FC predicted intelligence in younger adults, but not personality traits of neuroticism, consciousness, extraversion, and agreeableness. There are several possible reasons behind the weaker predictive power of resting-state FC for our emotional memory measures.

The first possibility might be a low reliability of emotional memory measures. A recent study reported that the emotional memory enhancement effect had a very low test–retest reliability when the same participants were tested twice over a delay of 10 weeks^42^ presumably due to the correlation between emotional and neutral memory measures and low between-subject variability in these subtraction scores^41^. In the Cam-CAN data, there were strong correlations between emotional and neutral memory measures; positive and negative object memory scores were highly correlated, *r*(301) = 0.90, *p* < 0.011; and neutral object memory performance was also highly correlated with both positive, *r*(301) = 0.89, *p* < 0.001, and negative memory performance, *r*(301) = 0.88, *p* < 0.001. The associative valence memory performance also showed high correlations between positive and negative conditions, *r*(301) = 0.77, *p* < 0.001, between neutral and negative conditions, *r*(301) = 0.85, *p* < 0.001, and between positive and neutral conditions, *r*(301) = 0.84, *p* < 0.001; although the magnitudes of correlation were weaker for the background memory, it still showed moderate correlations between negative and positive *r*(301) = 0.41, *p* < 0.001, between negative and neutral *r*(301) = 0.35, *p* < 0.001, and between positive and neutral conditions *r*(301) = 0.39, *p* < 0.001. Such strong correlations could have resulted in low reliability for our dependent variables (i.e., the emotional memory enhancement effect and the positivity bias score) that were derived by subtracting one from another highly correlated variable^64^. Thus, our failure to predict emotional memory measures may have been driven by the limited reliability of the measures.

The second possibility concerns our dependent measures. In our main analysis, we used memory performance for neutral objects learned with emotional backgrounds as the key dependent variable. Thus, the dependent measures were not about emotional items themselves but more about the effects of emotion (induced by the background images) on memory for nearby neutral information (i.e., neutral objects presented with the background images). Previous research has repeatedly shown that while emotional items are preferentially remembered better than neutral items in many situations, the effects of emotional items on nearby neutral information are more complex^7,65,66^; such that emotion sometimes enhances memory for nearby neutral information but sometimes impairs memory for nearby neutral information^66–69^. These findings point to the likelihood that resting-state FC has low prediction power for individual differences in memory for neutral items nearby emotional items (arguably due to the complex nature of the effects) but may be able to predict individual differences in memory for emotional items themselves. To address this possibility, we performed exploratory analyses on two additional memory measures that are more about emotional background images (i.e., valence and the content of a background image associated with each neutral object). However, once again, resting-state FC failed to predict individual differences in these two measures. Thus, resting-state FC does not seem to reliability predict individual differences in the effects of emotion on memory, irrespective of whether memory concerns emotional items per se or nearby neutral information.

Nevertheless, it is important to note that in the Cam-CAN project, the two measures of the emotional background images were not independent from the object memory measure; participants were given a chance to answer the valence and the content of a background image associated with each neutral object only when they recognized the neutral object as studied (see “Methods”). Thus, it is possible that resting-state FC can predict individual differences in emotional memory in other tasks (e.g., a simple recognition test; a free recall without constraints of associated object memory). Future research needs to address this issue.

Third and relatedly, the current study used performance in the memory test performed 10 min after the encoding session. However, previous research has suggested that the effects of emotion on memory are due to long-term consolidation effects^19^; thus future studies with long-term memory measures obtained after consolidation may obtain a different result. Furthermore, in the Cam-CAN project, the resting-state BOLD signals were obtained on a different day from the emotional memory task. Thus, the design could be particularly vulnerable to the low reliability of our emotional memory measures^42^. It is therefore possible that resting-state BOLD signals have stronger predictive power for emotional memory measures when they are obtained on the same day.

Fourth, recent research points out that FC derived from 5 to 10 min of resting-state data have low reliability to detect reliable individual differences^70–74^. Given that the Cam-CAN project has a relatively short resting-state data, the lack of significant effects in the present study may have been due to the low reliability of FC analysed in the present study. Future research needs to address this issue with data from longer resting-state scans. Likewise, recent research suggests that FC derived from task-state fMRI scans can enhance predictions of individual differences^74^. Therefore, future research could also combine using task-state functional connectivity and resting-state in order to achieve stronger predictability^75^.

Fifth, we had a relatively large sample size; in fact, our total sample size (n = 303) is sufficient to detect a relatively small sized correlation (see “Methods”). However, our sample size was modest after splitting participants into three age groups^57,76^, which could have resulted in the failure to predict emotional memory measures by resting-state FC. On the one hand, even with this same sample size, we still found that intelligence in younger adults was predicted by resting-state FC as observed in previous studies^36,49,77–79^. Yet, our sample size may not have been large enough to address the heterogeneity within older adults^80^. In addition, participants only had one resting-state session, which may have resulted in higher noise and lower prediction power than combining two or more sessions^36^. Future research needs to use a larger sample combined with multiple resting-state sessions and address the effects of resting-state FC.

Finally, although the main analyses investigated the effect across ages, it is notable that the preprocessing methods and predictive models used may be most appropriate for younger adults as the methods were developed and used primarily for a younger adult sample^36^. Unplanned exploratory analyses showed that intelligence was successfully predicted from resting-state FC in younger adults but not in middle-aged or older adults. Yet, as described earlier, age was predicted by resting-state FC successfully for participants from different age groups in this study. Such results for age were consistent with other findings^49^, suggesting that our analysis and denoising method was appropriate. The alternative reason behind the failure of predictions for middle-aged and older adults concerns the effects of age on individual differences. Previous longitudinal studies have suggested heterogeneity within older adults in their cognitive performance, brain structure and its functioning^80,81^. Thus, the age-related increases in the heterogeneity may have made it difficult for us to predict cognitive measures in middle-aged or older adults relative to younger adults. In line with this idea, a recent large-scale study including 711 older adults also found no association between cognitive performance and resting-state FC^63^ (but see Ref.^82^). Future research needs to take into account the effects of age on heterogeneity within participants.

In summary, the present study used a machine learning approach (which allowed us to select the most informative connections across the whole brain rather than relying on a priori selected regions) in predicting individual differences in emotional memory measures. While models derived from resting-state FC predicted age (for all participants) and intelligence for younger adults, they did not reliably predict the emotional memory enhancement effect and the positivity bias in memory for any age group. The results suggest the neural basis of individual differences in the emotional memory enhancement effect and positivity bias may not be meaningful or large enough to be predicted from resting-state FC. The results are in line with recent findings on low-reliability of the emotional enhancement effects in memory^42^, suggesting that more research should be done on the viability of the emotional enhancement effect and positivity bias as stable traits. Our results also support the use of an existing pipeline^36^ to denoise and predict traits at least for adult participants. Future research would be able to use this pipeline to minimise bias in choosing methods based on the results obtained (p-hacking)^76^.

## Methods

### Cam-CAN database

Data used in the preparation of this work were obtained from the CamCAN repository (available at http://www.mrc-cbu.cam.ac.uk/datasets/camcan/)^44,45^. A total of 306 participants, aged 18–87, have completed the structural MRI brain scans, resting-state fMRI scans, the emotional memory test and the intelligence test in the Cam-CAN dataset. Two participants were completely missing signal in significant portions of the cerebellum and the brain stem leading to errors in preprocessing. One participant had an incomplete resting-state fMRI scan lasting less than the database’s acquisition time of 8 min and 40 s. Therefore, the final sample size included 303 participants (N = 155 females; 18–87 years, *M*age = 54.3, *SD* = 18.1) who had structural and functional resting-state brain scans, behavioural measures on emotional memory, and intelligence scores. The data analysed in this study was the Cam-CAN consortium which has gained ethical approval from the Cambridgeshire 2 (now East of England-Cambridge Central) Ethics Committee. We did not perform a formal power analysis; The power computation for prediction *R*^2^ is not established because (1) there is no single true data generation model corresponding to a specific *R*^2^ value and (2) the true data generation model also varies depending on the algorithm (e.g., random forest, elastic net). But the sensitivity analysis suggests that our total sample size (n = 303) is sufficient to detect a relatively small sized correlation (r) of 0.16 at 80% statistical power with alpha = 0.05^83^. After splitting the sample into three age groups, the sample sizes were sufficient to detect a correlation (r) of 0.30 in younger adults, (r) of 0.28 in middle-aged adults, and (r) of 0.25 in older adults, with 80% power and alpha = 0.05.

### Emotional memory task

The memory task in the Cam-CAN database consisted of 120 trials, presented in two blocks^46^. In brief, every trial started with presentation of a background image for 2.5 s; the background was either positive, negative or neutral. Participants then saw a neutral object superimposed on the background for 7.5 s, during which they were asked to link the item and background by mentally creating a story that combines them. Participants performed a surprise memory test 10 min later.

During the memory test, participants were shown an object and asked to indicate whether or not it had been shown during the study phase (i.e., object memory). For objects indicated as ‘shown’, participants were asked to identify the valence of the background on which the object was superimposed (i.e., associative valence memory), then describe the background scene (i.e., background memory). Participants’ responses to the background memory test were coded to reflect whether participants described correct details, correct gist, incorrect information or no responses were given. The test had 160 trials (120 trials with old stimuli and 40 trials with new stimuli).

The current study used the d’ measure of discriminability^84^ for the object and the associative valence memory. For the background memory, we computed the proportion of trials where participants could correctly recalled gist. For all memory scores, two memory variables were created: the emotional enhancement effect and the positivity bias. The emotional enhancement effect was obtained by subtracting performance in the neutral condition from the average performance in the positive and negative conditions. The positivity bias measure was computed by subtracting performance in the negative condition from performance in the positive condition.

### Intelligence

The Cam-CAN database included a fluid intelligence test, the Cattell Culture Fair Scale 2 Form A^85^. The test has four subsets of nonverbal intelligence tests. A principal component analysis was performed on the scores of the four subsets to get one composite score of intelligence.

### MRI data acquisition

MRI scans were acquired using 3 T Siemens TIM Trio scanner^45^. Structural T1-weighted images were acquire using the 3D MPRAGE sequence: repetition time (TR) = 2250 ms, echo time (TE) = 2.99 ms, Inversion Time (TI) = 900 ms, flip angle = 9 degrees, GRAPPA acceleration factor = 2, resolution 1.0 mm isotropic*.* Every participant had one resting-state fMRI scan with an acquisition time of 8 min and 40 s, and a total of 261 volumes. Resting-state BOLD fMRI scans had the following parameters: TR = 1970 ms; TE = 30 ms; flip angle = 78 degrees; slices = 32 of thickness = 23.7 mm; field of view (FOV) = 192 mmx 192 mm; voxel size = 3 mm × 3 mm × 4.44 mm.

### fMRI preprocessing

We initially processed the raw functional MRI (fMRI) data obtained from the CamCAN database using FMRIB Software Library (FSL)^86^. Preprocessing included deleting the first two volumes in every scan. Motion correction was then performed on the raw resting-state images using FSL MCFLIRT^87^ (6 degrees of freedom), where the timeseries were realigned to the middle volume. Three participants (aged 23, 38 and 40) showed translational movement of over 3 mm in one or more volume. We did not exclude participants based on motion cut-off. Field map distortion correction was then applied, before setting high pass filtering cut-off to 100 s, and performing nonlinear registration of brain-extracted T1 images to Montreal Neurological Institute (MNI) space using FSL FNIRT (12 degrees of freedom). Each participant’s T1 structural image was skull/neck stripped using the FSL’s brain extraction tool (BET) and then used to create participant’s specific masks for the white matter, grey matter and cerebrospinal fluid (CSF) using FSL FAST. Although Dubois et al.^36^ found stronger prediction results when using multimodal surface-based alignment and registration (MSM) compared with MNI, we refrained from using MSM as it excludes subcortical regions, which are relevant for emotional memory^16^.

We next applied the same denoising steps as included in ‘Pipeline A’ from Dubois et al.^36^ given that this pipeline had the best prediction performance in predicting personality traits in this study. The pipeline started by z-score normalization of each voxel’s signals. Voxels in the white matter and CSF were then detrended by regressing out the temporal drifts. Next, the mean signals of CSF and white matter voxels were computed and regressed out from grey matter voxels. Motion regression was then performed using translational and rotational and temporal parameters as covariates which were regressed out from the whole-brain through linear regression. Low-pass filtering was performed using a Gaussian kernel with standard deviation of 1 TR. Finally, grey matter voxels were detrended for temporal drifts, followed by a global signal regression. The preprocessing and denoising pipeline scripts used are publicly available (https://github.com/donakand/EmotionalMemory).

The denoised resting-state images were then segmented into 268 nodes^48^; for each node, we averaged signals in all included voxels for each timepoint to create timeseries for each parcel. A total of 45 participants had missing data in one or more brain nodes; these missing data were restricted to seven nodes: 51, 58, 60, 185, 189, 194 and 202, corresponded to the left and right temporal lobes, located close to the surfaces of the brain^48^. To keep as many participants as possible, these seven nodes were excluded from the analysis. A connectivity matrix was created by correlating parcels’ time-series using Pearson’s correlation. The connectivity matrix consisted of 33,930 edges (connections) per participant.

### Machine learning analyses

Our main machine learning analyses used methods described in Dubois et al.^36^ Four separate analyses were carried out for different outcome variables: (a) the emotional enhancement effect, (b) the positivity bias, (c) intelligence and (d) age (as exploratory analysis that we did not pre-register). In all models, we included the connectivity from 33,930 edges as predictors. In addition, age, gender, handedness, and intelligence were used as control variables in the emotional enhancement effect and positivity bias analyses. Age, gender and handedness were controlled in the analysis of intelligence. Gender, handedness and intelligence were controlled for in the analysis of age. Similar to Dubois et al.^36^, the effects of these control variables were regressed out from the outcome measure using multiple linear regression before running a subsequent machine learning analysis. One participant was missing handedness information. The handedness for this participant was replaced by the median handedness value. Motion parameters were not used as control variables in the machine analyses, as motion correction and motion regression were applied to the resting-state scans during preprocessing and denoising (see Supplementary Table 1 for analyses including motion as a control variable). Motion was calculated as the mean translational realignment value. Motion was weakly correlated with age (r = 0.12, *p* = 0.03) and intelligence (r = − 0.18, *p* = 0.002), but not significantly correlated with the emotional enhancement effect (r = − 0.04, *p* = 0.45), positivity effect (r = − 0.05, *p* = 0.41), or intelligence in younger adults (r = − 0.20, *p* = 0.06).

For each machine learning analysis, a filtering approach was taken. Only the edges with correlations with the predicted variables with *p* value < 0.01 were included from the analyses. Next, we generated an Elastic Net model, implemented with Scikit Learn in python version 0.19.2^88^. Similar to Dubois et al.^36^ to choose optimal parameters, the model’s alpha value was tuned using a grid search of the parameter space, and a three-fold nested cross-validation. The Elastic Net mixing parameter L1 was set as 0.01. However, we also performed exploratory analyses where L1 was chosen through three-fold nested cross-validation. The model was trained and tested using a leave-one-out cross-validation. The model was evaluated using *R*^2^. As in Dubois et al.^36^, in the current study *R*^2^ was not the square of the correlation coefficient, but rather determined using Eq. (1). Therefore, *R*^2^ could take negative values in case of squared sum of errors larger than that of the null model—a horizontal line through the mean. As in Dubois et al.^36^, models were also evaluated on the normalized root mean squared deviation (nRMDS), which is the square root of the ratio of the standard deviation of residuals divided by the standard deviation of the observed values, and can be obtained directly from the *R*^2^, as in Eq. (2).

1 $${R}^{2}=1-\frac{{\sum }_{i=1}^{n}{\left({y}_{i} -\widehat{{y}_{i}}\right)}^{2}}{{\sum }_{i=1}^{n}{\left({y}_{i}-\overline{y }\right)}^{2}},$$

2 $$\mathrm{nRMSD}= \sqrt{1-{R}^{2}}.$$

To evaluate these results against a null hypothesis, under which the data is not predictive of our variables, and obtain a *p* value, we generated a null distribution by generating 1000 shuffled permutations of the memory scores in the dataset. We ran our models on every shuffled dataset. The one-tailed *p* value of the (actual) dataset model was then calculated by computing the number of permutations for which *R*^2^ was larger than the dataset’s model, divided by 1000.

To assert the specificity of the results obtained from our preregistered methodology, we conducted further analyses using alternative methods. The exploratory analyses retained the same control variables as our main analyses and were also evaluated using *R*^2^. The main analyses were run again while only changing the cross-validation from leave-one-out to 10-Fold cross-validation. This is due to a recent argument suggesting more robustness with k-Fold cross-validation in neuroimaging^57^. In keeping with the original methodology by Dubois et al.^36^, we set up the Elastic Net model using a proportion of L1 regularization of 0.01. However, other studies have obtained good prediction using larger L1 ratios^52,53^, or pure Lasso^54–56^ (L1 = 1). Therefore, we ran the main analyses again, tuning the L1 parameter in a nested threefold cross-validation from values: 0.1, 0.5, 0.7, 0.9, 0.99, 1. The filtering threshold applied before all prediction analyses, was set at 0.01. To check whether the threshold impacted the findings, exploratory analyses were run to predict intelligence for younger adults using filtering thresholds of 0.02, 0.03, 0.04, and 0.05. The highest R-square was obtained using a filtering threshold of 0.05 (Supplementary Table 2). The main analyses were rerun using a filtering threshold of 0.05. Finally, the main analyses were replicated using a different learning algorithm, Random Forest, rather than regularized linear regression. Random Forest is one of the most robust algorithms and has been used in psychology^89–91^. The algorithm does not require assumptions of linearity or collinearity of variables, and has shown good reliability^92–94^. The maximum depth parameter was chosen from values: 5, 10, 20, 40, 50 using a nested threefold cross-validation. Several past studies have successfully used similar parameters to implement Random Forest models in psychological and neuroimaging studies^58–61^.

Further analyses investigated whether the null findings were influenced by the exclusion of seven nodes which had missing data from the analyses. The main analyses were rerun while including all the nodes, resulting in 35,778 edges, for the 258 participants who did not have missing data in any node. The analysis followed the main analysis methods of filtering threshold at 0.01, Elastic Net mode with L1 = 0.01, and training the model through leave one-out cross validation.

## Supplementary Information

Supplementary Tables.

## Data Availability

Data used in the preparation of this work were obtained from the CamCAN repository (available at http://www.mrc-cbu.cam.ac.uk/datasets/camcan/). Users must agree to the terms and conditions and submit an application to access the CamCAN database. The dataset generated and analysed during the current study is available in the OSF repository, https://osf.io/bm98y/files/osfstorage.
