# Supplementary material for: Resting-state functional connectivity does not predict individual differences in the effects of emotion on memory
Source: Sci Rep. 2022 Aug 25;12:14481. doi: 10.1038/s41598-022-18543-8 (PMC9411155; doi:10.1038/s41598-022-18543-8)
Supplement: Supplementary file 1 — Supplementary Tables. [file 41598_2022_18543_MOESM1_ESM.docx]

**Supplementary Table 1. Prediction results when motion (mean translational realignment value) was added as a control variable in the prediction pipeline**

| **Dependent Variable** | **Ages** | ***r*** | ***R*^2^** | **nRMSD** |
| --- | --- | --- | --- | --- |
| Emotion enhancement effect | All Ages | -.14 | -.34 | 1.16 |
| Positivity bias | All Ages | .03 | -.19 | 1.08 |
| Intelligence | All Ages | .06 | -.12 | 1.06 |
| Age | All Ages | .44 | .19 | .90 |

**Supplementary Table 2. Model prediction results for intelligence in younger adults, using different filtering thresholds**

| **Dependent Variable** | **Ages** | **Filtering Threshold** | ***r*** | ***R*^2^** | **nRMSD** |  |
| --- | --- | --- | --- | --- | --- | --- |
| **Intelligence** | | | | | |  |
|  | Younger adults | 0.01 | .38 | .14 | .93 |  |
|  | Younger adults | 0.02 | .34 | .10 | .95 |  |
|  | Younger adults | 0.03 | .35 | .10 | .95 |  |
|  | Younger adults | 0.04 | .37 | .11 | .94 |  |
|  | Younger adults | 0.05 | .37 | .12 | .94 |  |
